# Biofertilizer based on halotolerant microorganisms promotes the growth of rice plants and alleviates the effects of saline stress

**DOI:** 10.3389/fmicb.2023.1165631

**Published:** 2023-06-09

**Authors:** Shiping Shan, Zhongwei Wei, Wei Cheng, Dongxia Du, Dianfeng Zheng, Guohui Ma

**Affiliations:** ^1^Hunan Institute of Microbiology, Changsha, Hunan, China; ^2^State Key Laboratory of Hybrid Rice, Hunan Hybrid Rice Research Center, Changsha, Hunan, China; ^3^Guangdong Ocean University, Zhanjiang, Guangdong, China

**Keywords:** salinized field, microbial remediation, biofertilizer, microbial diversity, soil electrical conductivity

## Abstract

Long-term soil salinization easily contributes to soil hardness, soil nutrient imbalance, and soil microbial diversity reduction, resulting in low rice yields in the salinized fields, and microbial remediation is one of the important measures to improve salinized soil. To verify the effect of biofertilizer based on halotolerant microorganisms on promoting rice growth and alleviating saline stress, this study discussed the effects of biofertilizer on soil microbial diversity and community structure and analyzed the correlation between the formation of microbial community structure and soil nutrient factors in the salinized field. The result, in comparison with applying inorganic fertilizer (referred to as CK), showed that notably increased soil available nitrogen, available phosphorus, available potassium, and rice paddy yield (*p* < 0.05) and significantly decreased soil electrical conductivity (*p* < 0.05) were achieved *via* biofertilizer (referred to as G2). Additionally, the application of biofertilizer contributes to the increase in soil microbial diversity and reorganization of microbial community structure, and through the analysis of linear discriminant analysis effect size, a notable difference in relative abundance was found in 13 genera, 6 families, and 3 orders between the control group and experimental groups (*p* < 0.05), and by linear discriminant analysis, *Desulfomonas* was further identified as the differentiated indicator. The redundancy analysis showed that available phosphorus and cation exchange capacity were the key environmental factors that affected microbial community structure and composition. Through bacterial functional prediction, increased rhizosphere soil bacterial metabolism, enzyme activity, membrane transport, and other potential functions were achieved by applying biofertilizer. Therefore, the application of biofertilizer could significantly alleviate rice growth stress and increase nutrient supply capacity in saline soil. These findings provide theoretical support for soil microbial improvement technology in the salinized field.

## 1. Introduction

Soil degradation caused by salinization is an issue globally, and it is getting worse. Soil salinization, which causes soil hardness, disorders of nutrient-supplying capacity, and restrained plant growth, has become one of the main problems hindering agricultural productivity. It causes not only economic losses and unsustainable agricultural development but also poses a threat to world food security (Qadir et al., 2014; Liu et al., 2022). Report data show that worldwide salinized land is distributed to over 100 countries with a total area of 955 million ha, with 99.1 million ha in China (Wang et al., 2017; van Zelm et al., 2020). It is predicted that, by the year 2050, almost 700 million ha of worldwide arable land will be affected by salinization (Wang et al., 2003). Through the osmotic stress at early growth stage and the ion stress at late growth stage caused by saline paddy fields, the viability of crops is affected (Lokhande et al., 2013; Jesus et al., 2015). Therefore, one of the major problems to be resolved in China is the improvement and reasonable utilization of the salinized land.

Currently, physical, chemical, and biological measures are used as the main tools to improve the salinized fields, among which, microbial remediation is an environment-friendly, economical, and effective way to reach salinization improvement (Meena et al., 2019). Soil microorganisms are the key biological factors affecting plant growth, especially the growth of halophytes in salinized soil (Yang et al., 2016). According to studies, soil microorganisms regulate ecosystem function and play an important role in biogeochemical cycles. In addition, the growth and activity of microorganisms are very sensitive to soil community structure formation and changes in the physical and chemical properties of soil (Deng et al., 2015; Wang et al., 2019b). It is found that there is a negative correlation between soil salinity and bacterial community assemblage. In addition, the increase in soil salinity contributes to decreased metabolism efficiency of microbial communities and microorganisms in the soil (Shao et al., 2019). Soil pH, salinity content, and enzyme activity are the main soil indexes used to evaluate amendment's effects on the soil microbial community and are closely related to the whole soil health condition of the saline-alkali ecosystem (Lu et al., 2020). Research showed that soil microorganisms could promote plant development and strengthen its resistance and the interaction between plants and soil microorganisms (Philippot et al., 2013; Zhou et al., 2021; Li et al., 2022). Although soil microorganisms are one of the indispensable environmental factors in the salinized field, the mechanism by which exogenous functional microbial communities reorganize soil microbial community structure in salinized soil and regulate the interaction between microorganisms and soil available nutrient factors has not been widely known. The aim of this study was to discuss the effects of biofertilizer based on halotolerant microorganisms on rhizosphere soil microbial diversity and community structure through the application of biofertilizer in salinized soil so as to reveal the microbiological mechanism of biofertilizer in promoting rice growth and increasing soil nutrient supply capacity. This study not only laid the foundation for the research and development of biological amendments but also provided support for the application of biological improvement measures on the salinized land in the future.

## 2. Materials and methods

### 2.1. Experimental location

The experiment was conducted at Xinzhouwei, Binhai New District, Yinhu Bay, Jiangmen City, Guangdong Province, at the seawater outlet of the Pearl River system. Instead of using fresh water, only fresh brine from seawater encroachment was used to irrigate the experimental field. The location, at the bottom of the land region of coastal plain, is topographically flat. The climate is a typical semitropical monsoon type, and the soil type is named Entisols (USDA Soil Taxonomy). The basic soil properties at the beginning stage of the experiment were as follows: pH, 7.00; salinity, 0.8%; electrical conductivity, 2.23 dS/m; soil organic matter, 21.1 g/kg; total nitrogen content, 0.91 g/kg; total phosphorus content, 0.85 g/kg; total potassium content, 1.84 g/kg; available nitrogen, 66.4 mg/kg; available phosphorus, 1.97 mg/kg; and available potassium, 174 mg/kg.

### 2.2. Components of biofertilizer

The biofertilizer based on halotolerant microorganisms is a special microbial fertilizer product for the salinized fields developed by our research group. The main active components of the product include a saline-tolerant synthetic microbial community, and its carrier materials are mainly organic materials, including crop straw and biological carbon, and inorganic materials, including phosphogypsum and sulfur sugar filter mud. The contents of N, P, and K are 4.0%, 7.5%, and 3.5%, respectively. The organic matter content is 40%, and the colony-forming unit number of biofertilizers was more than or equal to 50 million/g.

### 2.3. Experimental design

The one-cropping season rice variety Jingyou 007 was planted in the experimental field. The experimental groups (G1 and G2) and the control group (CK) were chosen for the experiment. By using different amounts of inorganic fertilizer for G1, G2, and CK, approximately equivalent N, P, and K content was reached for G1, G2, and CK (Table 1). The area of each experimental plot was set at 5 m × 6 m, and designed according to a random block group; single irrigation and single platoon were used in each district, and each treatment was repeated three times. Each treatment is numbered as follows: inorganic compound fertilizer for CK (CK1, CK2, and CK3); biofertilizer 2,250 kg/ha for G1 (GW1, GW2, and GW3); biofertilizer 3,000 kg/ha for G2 (GR1, GR2, and GR3).

**Table 1:** Names and applying amounts of the fertilizers for different groups.

**Name**	**Applying amount (kg/ha)**		
	**CK**	**G1**	**G2**
Biofertilizer	0	2,250.0	3,000.0
Urea	370.5	273.0	240.0
Potassium chloride	244.5	97.5	54.0
Superphosphate	1,219.5	300.0	255.0
N, P, K content	N:170.4 P:170.7 K:146.7	N:170.5 P:170.5 K:145.7	N:170.4 P:170.3 K:145.8

N, nitrogen; P, phosphorus; K, potassium.

### 2.4. Soil sample collection, analysis, and high-throughput sequencing

After the mature stage of rice, a soil collector (diameter: 50 mm) was used to randomly collect soil samples at a depth of 0–20 cm in each plot using the five-point sampling method. The 5 collected soil samples were mixed to obtain the soil sample for each treatment.

The alkali-hydrolyzed diffusing method was used to measure soil available nitrogen. The Mo-Sb colorimetric method was used to measure soil available phosphorus. FP6450 flame photometer was used to measure soil available potassium. Conductometry was used to measure salinity in the soil. The electrical conductivity (EC) was measured using a conductivity meter. The bichromate oxidation method was used to measure organic matter in the soil. The barium chloride method was used to measure CEC (Lu, 1999; Yan et al., 2021).

High-throughput sequencing was conducted by Majorbio Bio-pharm Biotechnology Co., Ltd. with Illumina HiSeq (San Diego, CA, USA) as the platform (Kõljalg et al., 2005; Quast et al., 2013). Original sequence data have already been uploaded to the NCBI sequence database (accession number: PRJNA891373) (Supplementary Table 1).

### 2.5. Statistical analysis

The SPSS software (version 19.0, Chicago, Illinois, USA) was used to conduct ANOVA and Duncan's test. The online platform Majorbio (https://cloud.majorbio.com/) was used to conduct bioinformatics analysis (Ao et al., 2020).

## 3. Results

### 3.1. Impacts of biofertilizer on rice yield

Based on data from our previous experiments on salinization improvement for the salinized fields (data unpublished), in order to increase rice yield and improve salinization in the field, the recommended application amounts of saline-tolerant biofertilizer are 2,250 kg/ha and 3,000 kg/ha, with 3,000 kg/ha being the optimal amount. The impacts of biofertilizer application amounts on rice yield and yield components varied (Table 2). Compared with CK, a yield increase of 12.91% was reached in G1 and 15.66% in G2. The result shows a remarkable yield increase in rice paddy in G1 and G2 through the use of biofertilizer. Furthermore, when comparing G1 with G2, G2 outyields G1 by 2.75%, which proves that increasing the application rate of biofertilizer could help increase rice yield. The yield increase of rice in G1 and G2 is attributed to the increase in yield components of rice, namely, effective panicles, total kernel number per panicle, setting rate, and 1,000-kernel weight. Moreover, the yield increase at 3,000 kg/ha of the application rate of biofertilizer was more significant than that at 2,250 kg/ha.

**Table 2:** Effects of biofertilizer on rice yield and yield components.

**Treatment**	**Effective panicles (10 thousand/ha)**	**Total kernel number per panicle**	**Setting rate (%)**	**1000-kernel weight (g)**	**Actual yield (kg/ha)**	**Yield increase (%)**
CK	380.3 ± 12.31a	88.58 ± 3.01a	92.17 ± 3.75a	26.04 ± 3.96a	7,210.1 ± 296.85b	-
G1	376.8 ± 8.76a	90.20 ± 5.02a	95.01 ± 3.00a	27.58 ± 2.70a	8,140.8 ± 375.75a	12.91
G2	383.2 ± 15.07a	93.36 ± 4.25a	94.05 ± 4.03a	27.32 ± 2.01a	8,339.4 ± 298.65a	15.66

The results were given as mean ± SD (standard deviation). Different lowercase letters within a column indicate significant differences at the *p* = 0.05 level among the different treatments.

### 3.2. Difference analysis of available nutrients in salinized soil

Compared with CK, significant increases in AN, AP, and AK were achieved in the soil of G2 (*p* < 0.05) (Table 3), while notable increases in AN and AP were reached in the soil of G1. The result shows that a remarkable increase in available nutrient content has been achieved through the use of biofertilizers in the salinized fields. Additionally, the performance of G2 is more evident than that of G1, which proves that added amount of biofertilizer could achieve a significant increase in available nutrient contents in soil. In comparison to CK, the contents of OM and CEC were also increased at varying degrees in the soils of G1 and G2. The OM in the soil is the main source of the nutrients and energy needed by the microbial vitality of the soil. Therefore, the increased content of OM in soil could provide a healthy living environment for the microorganisms in the soil. Furthermore, the increased content of CEC could strengthen the soil's capacity to preserve fertility, supply fertilizer, and provide buffering in soil.

**Table 3:** Analysis of soil physicochemical properties.

**Treatment**	**AN mg/kg**	**AP mg/kg**	**AK mg/kg**	**OM g/kg**	**CEC cmol(+)/kg**
CK	66.4 ± 1.98c	1.97 ± 0.12b	174 ± 5.29b	20.1 ± 0.81a	17.1 ± 0.35a
G1	81.6 ± 1.40b	2.65 ± 0.13a	177 ± 4.16ab	20.8 ± 1.05a	17.7 ± 1.15a
G2	101 ± 6.56a	2.54 ± 0.08a	186 ± 5.29a	20.7 ± 0.95a	17.8 ± 0.95a

AN, available nitrogen; AP, available phosphorus; AK, available potassium; OM, organic matter; CEC, cation exchange capacity. The results were given as mean ± SD (standard deviation). Different lowercase letters within a column indicate significant differences at the *p* = 0.05 level among the different treatments.

### 3.3. Dynamic difference analysis of soil electrical conductivity

**Figure 1:**
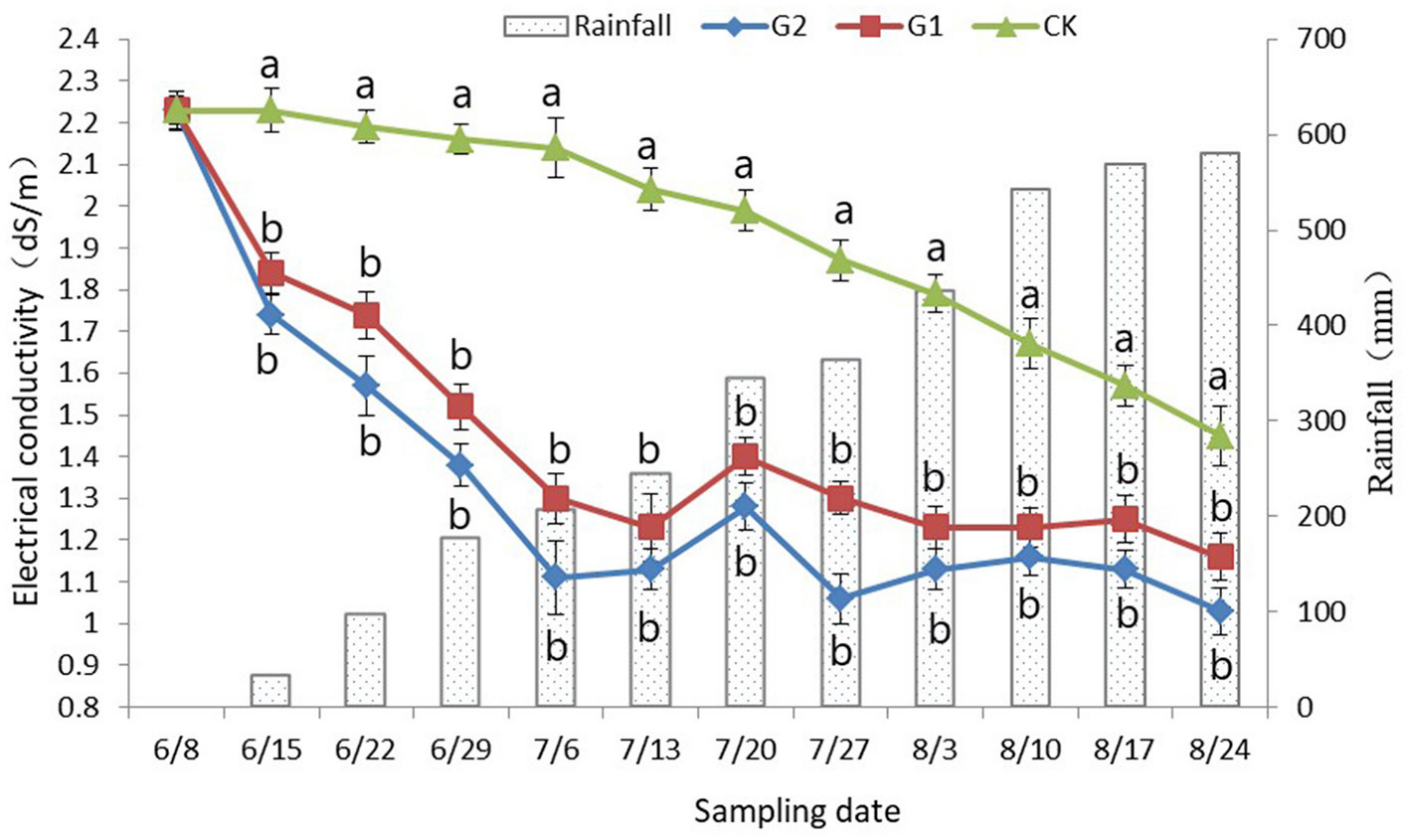
Dynamic changes of rhizosphere soil electrical conductivity with rainfall. Data were shown as means ± standard deviations. The letter following each value indicates the significance of the differences between values within a column in the alphabetic order (*p* < 0.05).

In comparison with CK, a significant decrease in the soil electrical conductivity was achieved in G1 and G2 (*p* < 0.05) (Figure 1). No changes in soil electrical conductivity were found at the early stage of the rice growth period in CK; however, at the late stage of rice growth, when it was affected by rainfall and rice root exudate, a slight decrease in the soil electrical conductivity was reached, and as the accumulated rainfall began to increase, the electrical conductivity reached its minimum at the maturing stage of rice. However, the electrical conductivity still stayed at approximately 1.45 dS/m (Figure 1). At the early stage of the rice growth period in G1 and G2, a sharp decrease in electrical conductivity in soil was achieved, and the electrical conductivity was maintained and stabilized at approximately 1.20 dS/m at the late stage of the rice growth period. In addition, the performance was more notable in G2. Therefore, not only a remarkable increase in soil nutrients was reached through the use of biofertilizer in the salinized field, but also a significant decrease in soil electrical conductivity was also achieved.

### 3.4. Difference analysis on bacterial diversity and richness indexes

Based on the classification level of species, analysis of bacterial community diversity and richness indexes of rice rhizosphere soil was conducted under different treatments in the salinized field (Table 4). Compared with CK, the Sobs diversity index, ACE index, and Chao1 richness index of soil bacteria in G1 and G2 were increased at varying degrees; the result was G2 > G1 > CK, among which that of G2 reached a significant level (*p* < 0.05). This indicates that sufficient biofertilizer is needed by the rice rhizosphere to regulate the bacterial community abundance in the soil so as to reach a level of significance. The Shannon diversity index of soil bacteria also increased in G1 and G2 at varying degrees, and when compared to CK, the result was G2 > G1 > CK.

**Table 4:** Analysis of bacterial community richness and diversity indexes.

**Treatments**	**Diversity index**		**Richness index**	
	**Sobs**	**Shannon**	**ACE**	**Chao1**
**Bacteria**				
CK	2,113 ± 510.67b	6.4 ± 0.36a	2,348 ± 596.51b	2,376 ± 584.26b
G1	2,570 ± 14.18ab	6.6 ± 0.22a	2,906 ± 239.090ab	2,919 ± 228.91ab
G2	2,841 ± 182.58a	6.7 ± 0.021a	3,308 ± 364.99a	3,310 ± 349.89a

The results were given as mean ± SD (standard deviation). Different lowercase letters within a column indicate significant differences at the *p* = 0.05 level among the different treatments.

**Figure 2:**
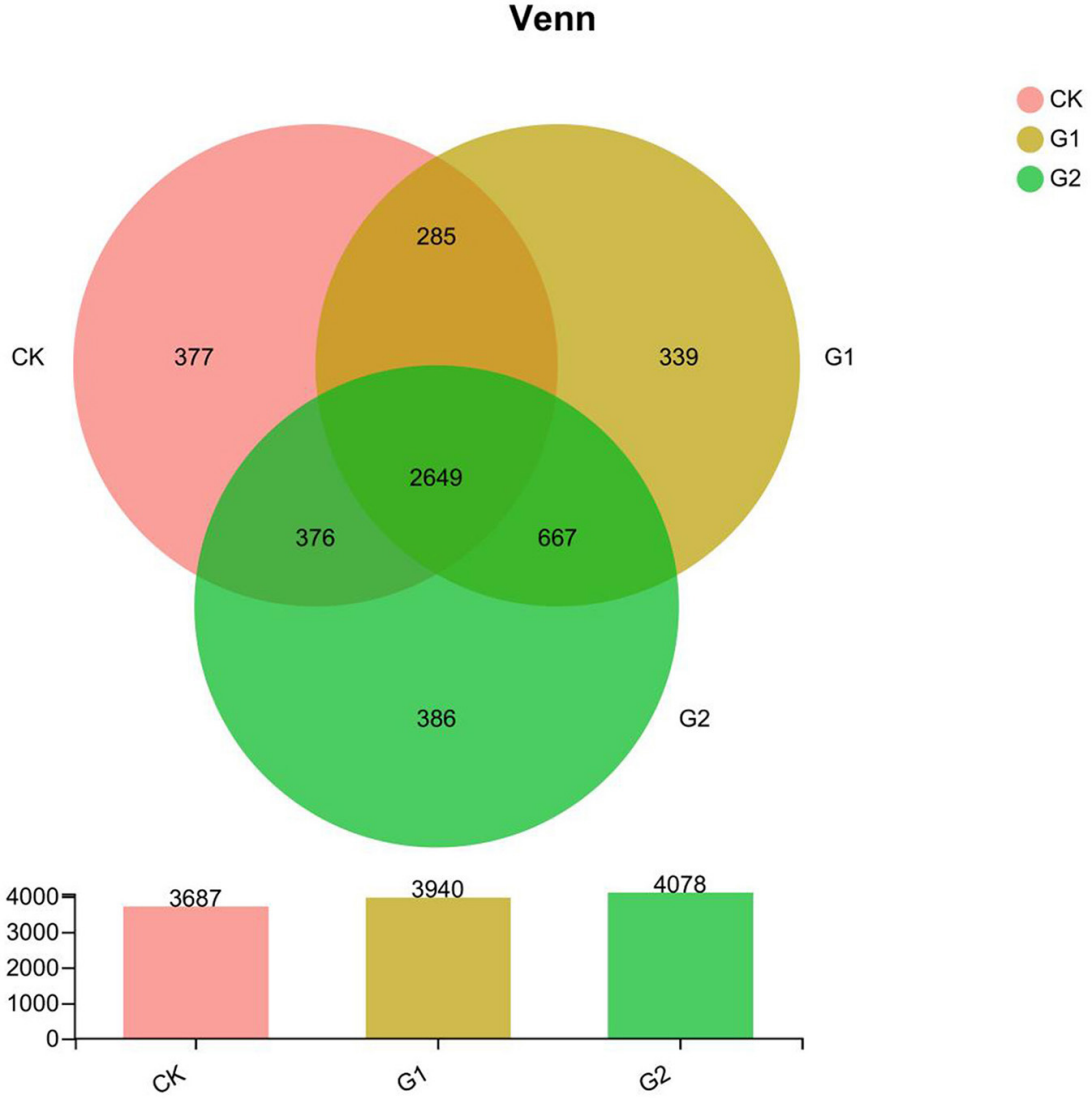
The Venn map at OTU level for the characteristic number of rice rhizosphere bacteria in different treatments. Values are the mean of three replicates.

Further studies on the diversity of soil bacterial community abundance indicated that a significant increase in bacterial community abundance was found in 3 areas, namely, total species, unique species, and shared species. According to the Venn diagram (Figure 2), the total species number at the OTU level of the rice rhizosphere bacterial community of CK, G1, and G2 is 11,705 (G2>G1>CK). Compared with CK, the increase in the species number of G1 and G2 over CK is 6.9 and 10.6%, respectively. The unique species number of CK, G1, and G2 are 377, 339, and 386, which reveal a notable difference. The unique species number of G2 is the highest. The total shared species number of CK, G1, and G2 is 2,649, among which G1+CK is 285, G2+CK is 376, and G1+G2 is 667, indicating a higher number of G1 and G2 than that of CK.

### 3.5. Analysis of soil bacterial community structure and composition

#### 3.5.1. Difference analysis of bacterial community formation

**Figure 3:**
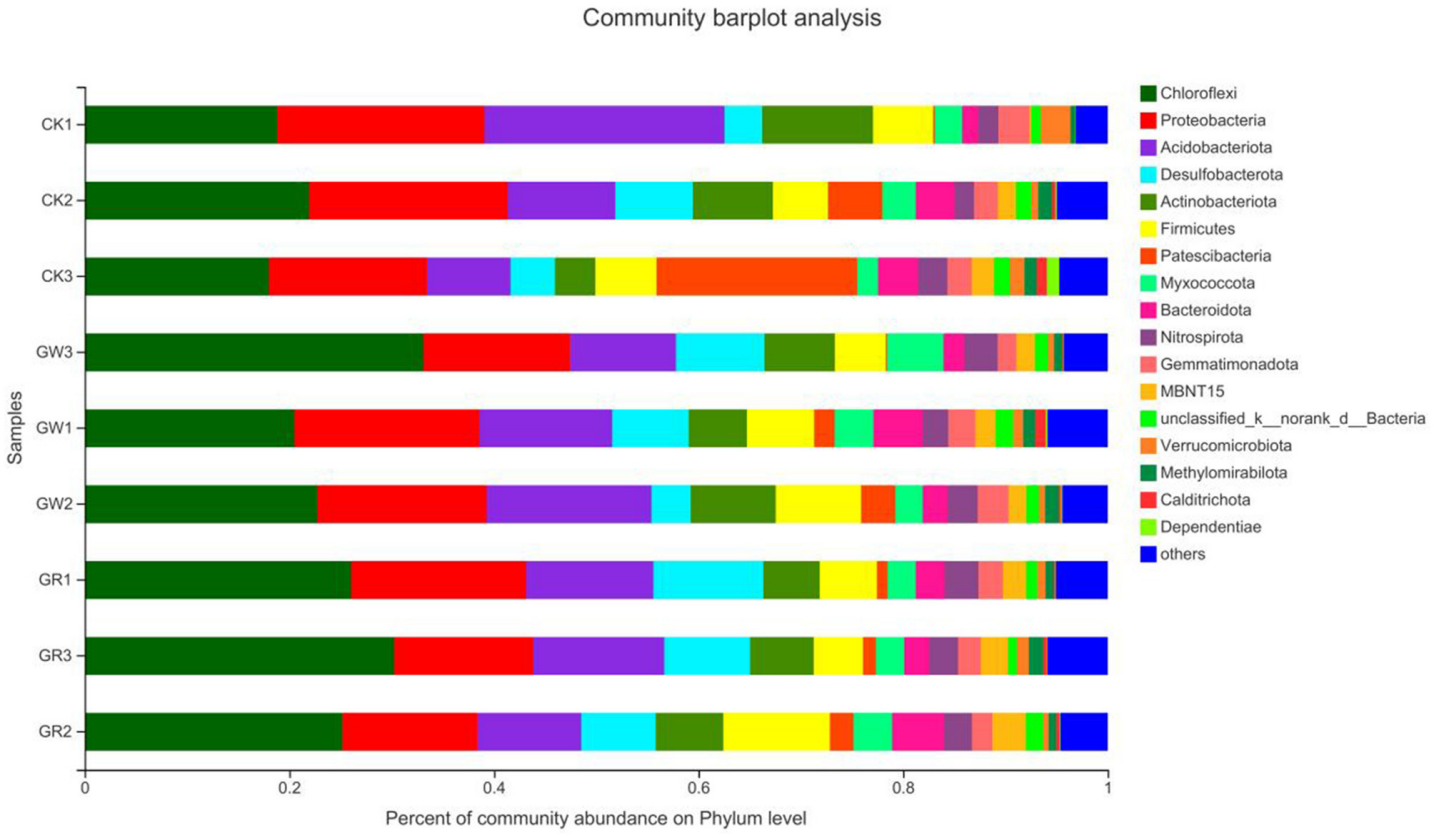
The species distribution at the phylum level of rice rhizosphere bacteria in different treatments.

The species distribution at the phylum level of rice rhizosphere bacteria under different treatments is shown in Figure 3. Additionally, the proportion of *Chloroflexi* and *Desulfobacterota* is notably higher in G1 and G2 than that of CK. However, the proportion is more evidently increased in G2. Additionally, the proportion of other species is also notably higher in G2 than that of CK, indicating that among the unknown bacterial communities of G1 and G2, which have a higher proportion, rare species that can be found in the salinized fields may exist. These species need to be further isolated, purified, and identified by the spread-plate method.

#### 3.5.2. Similarity analysis over the bacterial community formation

**Figure 4:**
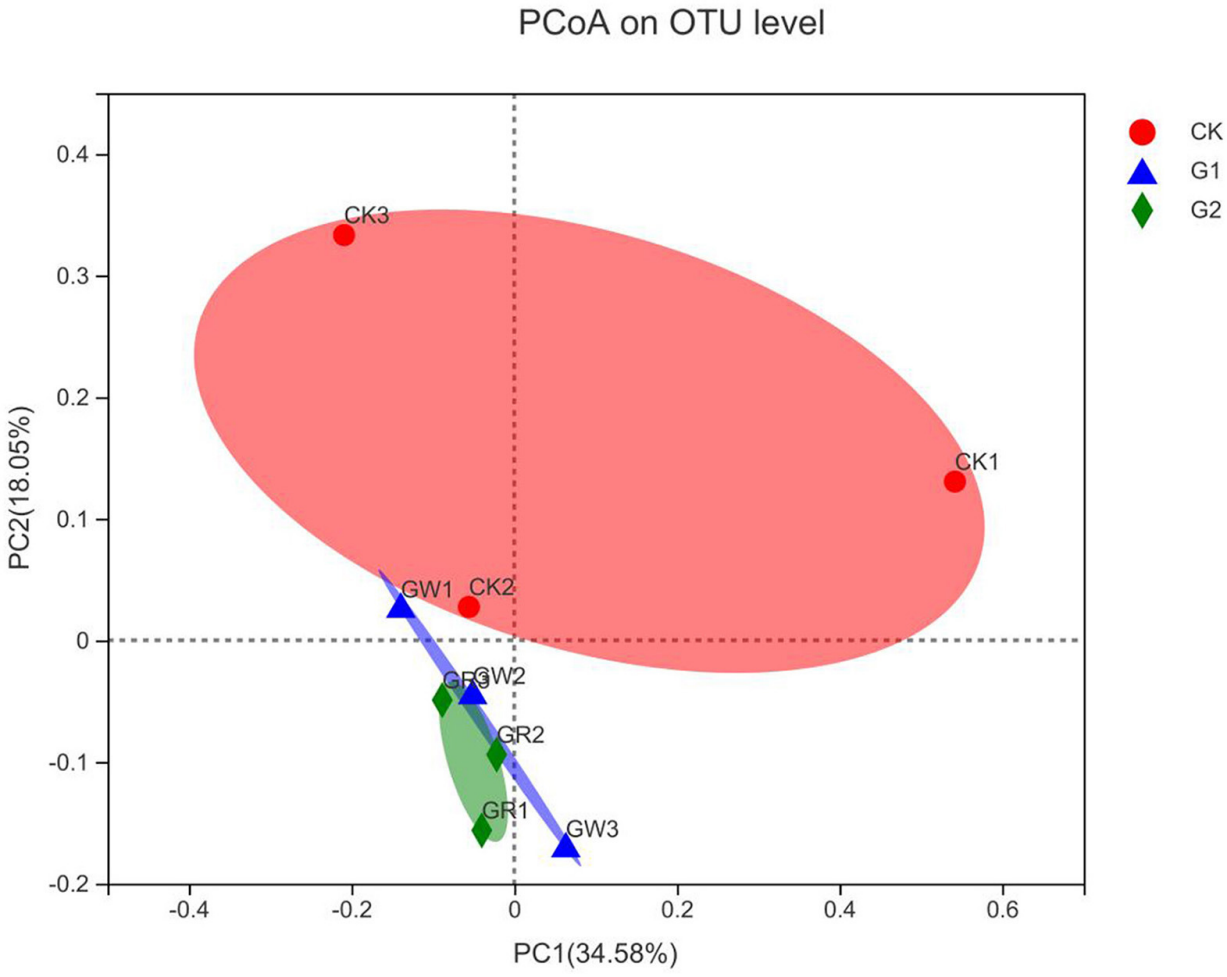
Bray-Curtis principal coordinates analysis of rice rhizosphere bacteria in different treatments.

The above studies revealed that notable impacts on bacterial community structure and abundance have been achieved in G1 and G2. To further analyze the similarity of bacterial community formation in different treatments, PCoA was conducted based on the Bray-Curtis distance of OTU level (Figure 4). The dots of different shapes are from samples of different treatments. The closer the two sample points are, the more similar the species composition of the two samples is. As shown in Figure 4, there is no overlap between CK, G1, and G2, and the sample dots are distant, indicating the rhizosphere soil microbial community was different between the control group and the experimental groups. The cumulative contributions of the X-axis (34.58%) and the Y-axis (18.05%) reached 52.63%, which was the main reason for the different compositions of microbial communities among samples in different experimental treatments.

### 3.6. Differential analysis of specific species

**Figure 5:**
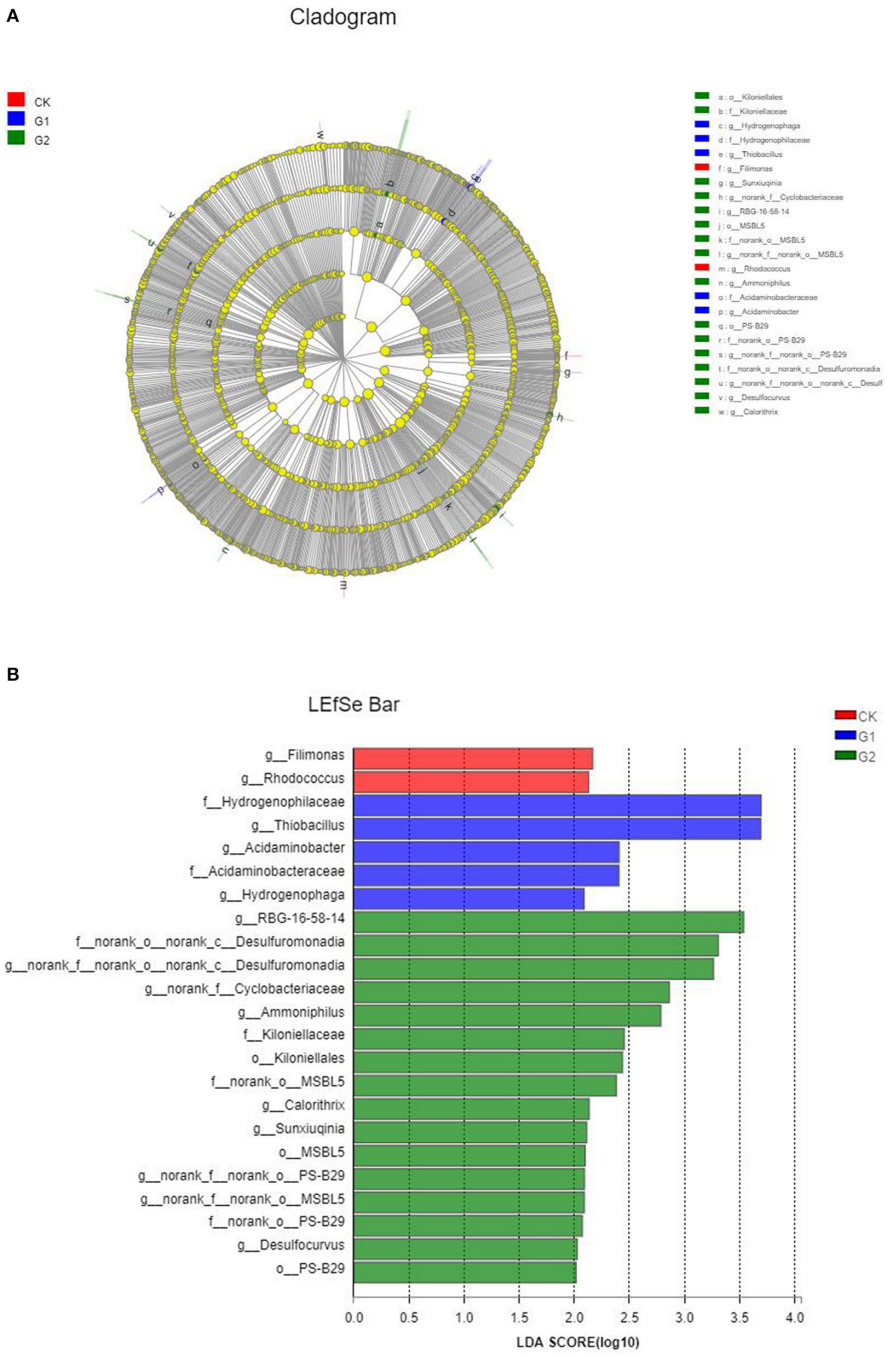
Cladogram **(A)** and histogram **(B)** of linear discriminant analysis effect size (LEfSe) analysis among the control group and the experimental groups.

Based on the above analysis, there are remarkable differences in terms of microbial species number, community structure, and abundance of soil samples from different treatments. However, are there significant differences among different species of microbial communities in different treatments? It remains unanswered. To find the answer to the question, a comparative analysis was conducted using LEfSe analysis, and in LEfSe analysis of different samples, the inside-to-outside of the concentric circle is phylum, class, order, family, and genus, respectively. In addition, the nodes represent specific bacterial communities from different treatments. The results indicated that the relative abundance difference of 13 genera, 6 families, and 3 orders in CK, G1, and G2 reached the level of significance (*p* < 0.05) (Figure 5A). The magnitude of the effect of the difference in abundance of each component (species) was further estimated by linear discriminant analysis (LDA). As shown in Figure 5B, the differential indicator species in the soil of CK are connected to the genera *Filimonas* and *Rhodococcus*; those of G1 are connected to the family of *Hydrogenophilaceae* and the genus *Thiobacillus;* and those of G2 are connected to the class *Desulfuromonas*. These species play important roles in affecting the differences among microbial communities' formation in soil samples in the salinized field.

### 3.7. Correlation analysis

**Figure 6:**
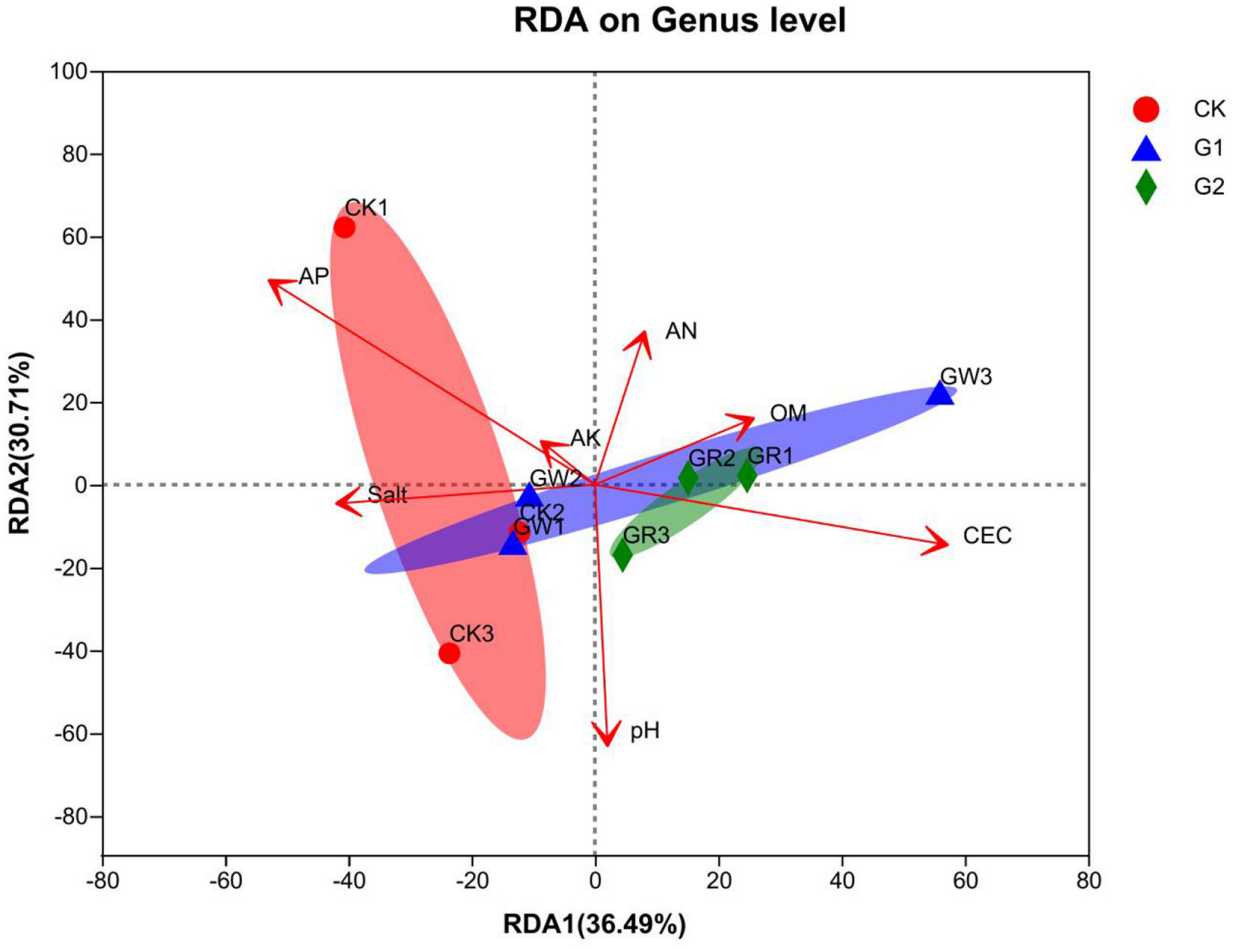
RDA analysis of the relationship between bacterial communities at the genus level and the soil physicochemical properties.

There is a close relationship between soil bacterial community structure and soil environmental variables. In this study, taking AN, AP, AK, OM, pH, CEC, and salinity as environment variables, redundancy analysis (RDA) of soil bacterial community structure formation and soil environment variables was conducted at the genus level to explain the correlation between soil bacterial community structure and soil environment variables. From RDA (Figure 6), the length of the arrow line of environment variables represents the interpretation quantity of environment variables as factors in species data. The longer the arrow line, the bigger the quantity interpretation. The angles among these arrow lines represent the correlation. Acute angles represent a positive correlation, and obtuse angles represent a negative correlation. In addition, the cumulative contribution of the X-axis (36.49%) and the Y-axis (30.71%) reaches 67.2%, which explains the correlation of soil bacterial community structure and soil environment variables among the samples of different treatments. According to the RDA results, AP (r^2^ = 0.6396, *p* = 0.045) and CEC (r^2^ = 0.6123, *p* = 0.042) are the main environmental variables affecting the formation of soil bacterial community structure in the salinized fields.

**Figure 7:**
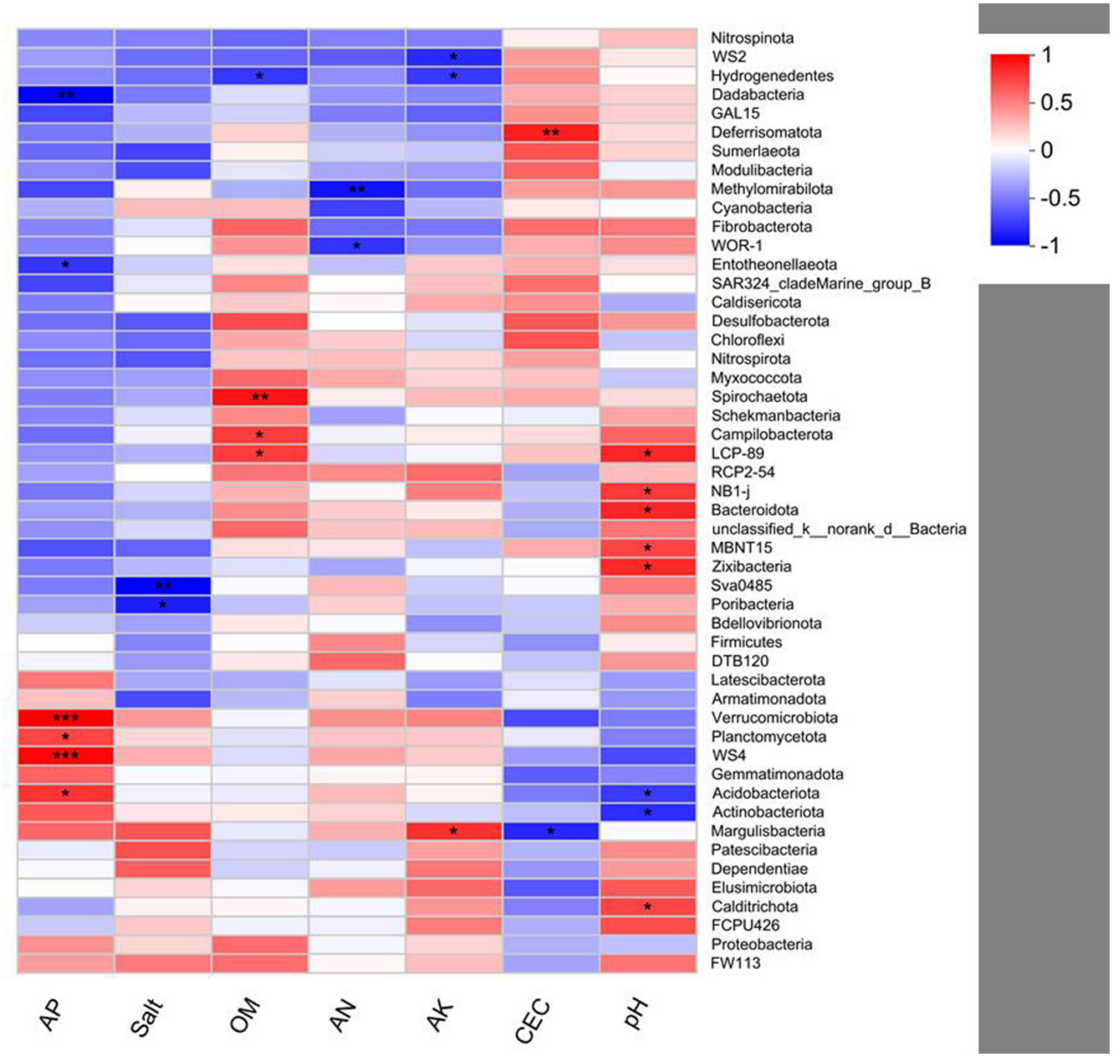
Pearson's correlation analysis between the bacterial community and soil variables. *p < 0.05; **p < 0.01; ***p < 0.001.

Pearson's correlation analysis of bacteria and soil environment variables indicates that the relative abundances of *Verrucomicrobia (p* < 0.001), *Planctomycetota* (*p* < 0.05), *Acidobacteriota* (*p* < 0.05), and WS4 (*p* < 0.001) are positively correlated with AP. The relative abundance of *Deferrisomatota* (*p* < 0.01) is positively correlated with CEC. The relative abundances of LCP-89 (*p* < 0.05), *Caldirichota* (*p* < 0.05), NB1-j (*p* < 0.05), *Bacteroidota* (*p* < 0.05), MBNT15 (*p* < 0.05), and *Zixibaceria* (*p* < 0.05) are positively correlated with pH. The relative abundances of *Spirochaetota* (*p* < 0.01), *Campilobacterota* (*p* < 0.05), and LCP-89 (*p* < 0.05) are positively correlated with OM. In addition, the relative abundance of *Margulisbacteria* (*p* < 0.05) is positively correlated with AK (Figure 7).

### 3.8. Functional analysis of soil bacterial community

**Figure 8:**
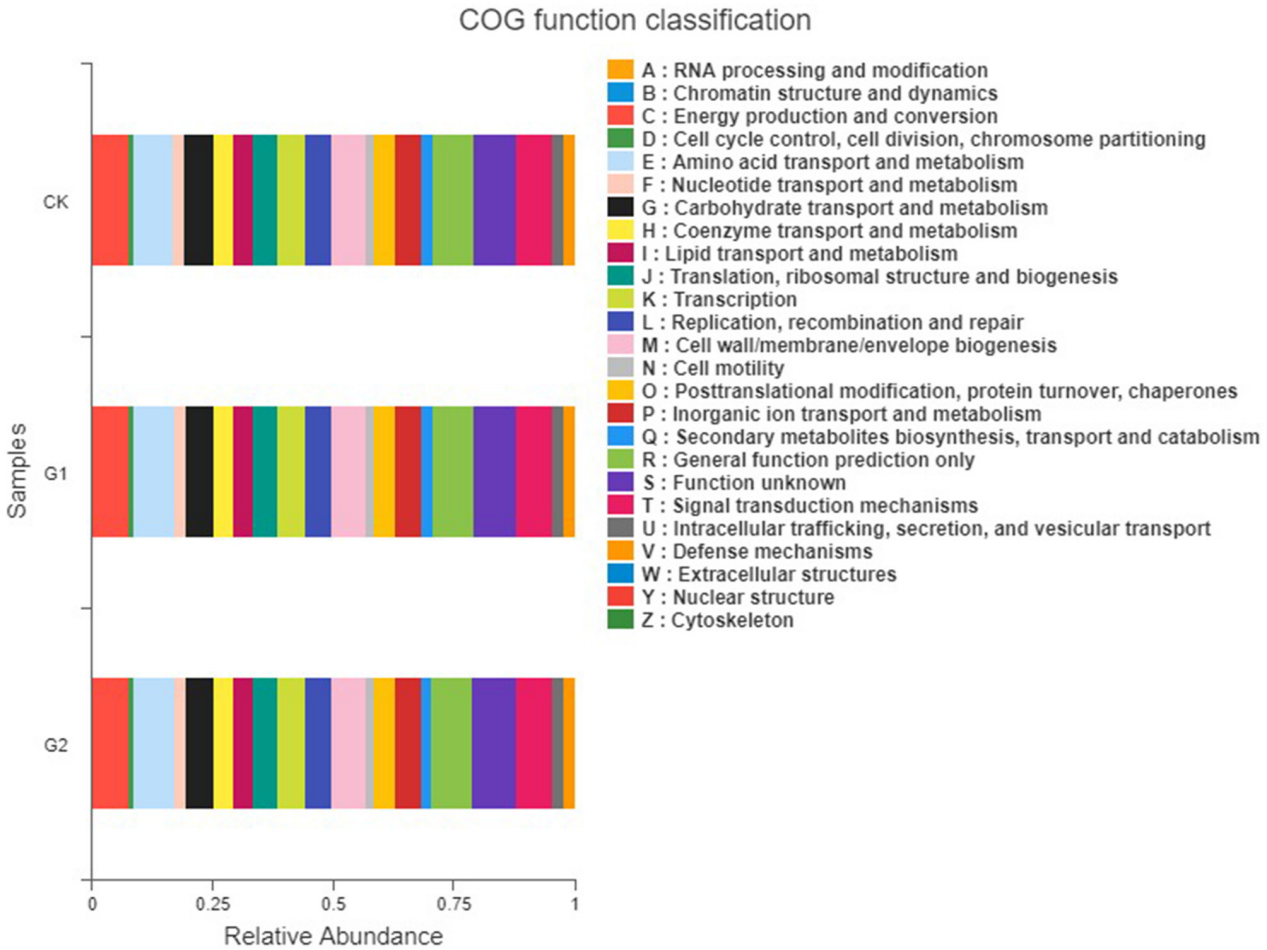
COG functional classification of rice rhizosphere soil in different salinized fields.

To understand the function of the rice rhizosphere soil bacterial community of the different treatments, the PICRUSt was used to predict the function of the bacterial community (Figure 8). Using predictive analysis, three levels of information on rice rhizosphere soil metabolic pathways and their abundances were obtained (Supplementary Tables 2–4). There are 7 metabolic pathways at Pathway level 1, 41 metabolic pathways at Pathway level 2, and 298 metabolic pathways at Pathway level 3. The metabolic pathways of rice rhizosphere soil bacterial community of all treatments are similar in a soil environment of salinity, but their abundance values are different. In first-grade metabolic pathways, apart from the unclassified pathways, there are 6 types of biologically functional metabolic pathways: cellular processes, environmental information processing, genetic information processing, human diseases, metabolism, and organismal systems, among which, metabolism, genetic information processing, and environmental information processing are main functions. Compared with CK, the three main functional abundances of G1 are increased by 15.5, 17.5, and 14.8%, and those of G2 are increased by 18.1, 19.6, and 19.3%, respectively. In the predictive analysis of second-grade metabolic pathways, it is found that the second-grade metabolic pathways consist of 41 sub-functions, including amino acid metabolism, carbohydrate metabolism, cellular processes and signals, energy metabolism, environmental adaptability, membrane transport, replication and cytothesis, and degradation of allogenic materials, among which cellular processes and signals, membrane transport, carbohydrate metabolism, amino acid metabolism, and cellular enzyme families of bacteria take a higher proportion. Compared with CK, the five main functional abundances of G1 are increased by 17.3, 14.3, 14.9, 15.3, and 16.3%, and those of G2 are increased by 19.6, 19.3, 19.2, 17.2, and 18.8%, respectively. The abundances of the main metabolic pathways of G1 and G2 are higher than those of CK, indicating that the application of the biofertilizer to the salinized field could strengthen the metabolic capacity of the soil bacterial community.

## 4. Discussion

Soil is the habitat for microorganisms and is critical to their survival and reproduction. It is generally acknowledged that soil is identified as salinized when the accumulation of water-soluble salt exceeds 0.1% on the surface or sub-surface of soil or the degree of alkalization exceeds 5% in the alkalized layer of soil (Haj-Amor et al., 2022). The excessive soluble salt contributes to not only reduced absorption of nutrients by crops but also physiological drought or even the death of plants (Marghoob et al., 2022). The soil microorganisms boast not only huge quantity and variety but also utmost adaptability, so that it can survive and play roles in many minor environments that are not suitable for other types of life. The soil microorganisms are involved in matter and energy circulation in nature and play an important role in improving salinity problems in the salinized fields (Wang et al., 2019a). Therefore, soil microorganisms are vital to improving salinized soil. Compared with other improvement technologies, the microbial improvement technology is more effective and environment-friendly, and as an important tool to realize green development of the coastal salinized field, it has become one of the research hotspots of soil improvement in saline-alkali land (Castaldi et al., 2023; Zhou et al., 2023).

The application of biofertilizer can effectively optimize the number of beneficial microorganisms in soil and make them become the dominant bacterial community. Compared with CK, significant increases in the number of bacteria were noted in G1 and G2 after applying biofertilizer, which is possible because the bacteria in soil are originally the main part of the microflora of ecosystems (van der Heijden et al., 2008; Wang et al., 2019b). However, the biofertilizer contains a large amount of synthetic bacterial community; as a result, not only the nutrient content was increased after applying biofertilizer, but also a significantly higher quantity of bacteria was achieved.

The Sobs index reflects α-diversity; the bigger the Sobs index is, the richer the microbial diversity is. The ACE and Chao1 indexes reflect the abundance of the communities in the samples; the bigger the Chao1 or ACE index is, the richer the communities are. The results showed that applying biofertilizer could increase the Sobs diversity index, ACE index, and Chao1 richness index of soil bacteria in the salinized field. However, there was no significant difference in the increase of G1 when the dosage was small. Only when the application amount reached a certain level (G2), did the difference in soil bacterial microbial diversity and richness index between different treatments reach a significant level (*p* < 0.05). It also showed that the synthetic microbial community in the biofertilizer played an important role in aspects including increasing microbial diversity of soil, changing the formation of soil bacterial community structure, and raising soil fertility.

To further characterize the dominant microflora of rice rhizosphere and its functions, this study analyzed the differences in the abundance of dominant microflora of rice rhizosphere between the control group and the experimental groups and further identified the differential indicator species between the groups by comparing the differences in the dominant microflora of rice rhizosphere.

According to the linear discriminant analysis (LDA) (Figure 5), *Desulfuromonas* was the dominant microflora in G2, indicating that this species played an important role in the difference in community composition between CK and G2 in the rhizosphere of rice in the salinized field. This kind of strain is subjected to sulfate-reducing bacteria and is an early anaerobic prokaryotic microorganism in the biosphere. It plays an important role in the biogeochemical cycle of sulfur elements, degradation of organic substances, reduction of metal ions, and so on, showing strong potential for microbial remediation. These strains can rapidly absorb and degrade the negative and positive ions in the salinized soil and fundamentally reduce the content of soluble inorganic salts in the soil. These strains can also reduce sulfate, sulfite, and thiosulfate in salinized soil to hydrogen sulfide and then eliminate sulfate anions. Meanwhile, through reduction, absorption, complexation, and precipitation of the excessive metal cations, the improvement of the salinized soil can be fulfilled. For further explanation, with the regulation of exogenous functional microorganisms, sulfate-reducting bacteria are gradually playing an important role. The application of biofertilizer changed the composition of bacterial community structure. To a large extent, the artificial bacterial community contained in biofertilizer realized the recombination of bacterial community structure by changing the dominant bacterial community.

PICRUSt is the most emerging bacterial community function prediction platform (Zhang et al., 2021). To investigate the effects of salt-tolerant biofertilizers on the function of the bacterial community in rice rhizosphere soil, HiSeq high-throughput sequencing was used for PICRUSt function prediction analysis. The results showed that the bacterial community of rice rhizosphere soil in the control group and experimental groups involved 6 metabolic pathways belonging to 41 subfunctions, showing functional richness.

In this study, compared with CK, the functional genes in the experimental groups with a higher proportion were all related to metabolism, membrane transport, and enzyme family, and the result is G2 > G1 > CK, indicating that biofertilizer had a greater impact on the activity of these functional genes during the growth of rice, among which G2 had the greatest impact. It indicated that adding more biofertilizers could further strengthen the activity of functional genes. In particular, carbohydrate metabolism and amino acid metabolism are closely associated with phosphorus and nitrogen cycling in plants, which is also the main reason for the significant increase in available nutrient content in G2. The optimized rhizosphere-dominant bacteria of rice play an important role through improved metabolism of functional genes and enzyme activity, resulting in promoted crop growth and increased crop yield (Wang et al., 2023).

## 5. Conclusion

In this study, Illumina Hiseq high-throughput sequencing was used to investigate the effects of biofertilizer on the bacterial community structure and functions in rice rhizosphere soil in the coastal salinized fields. The results showed that the application amount of 3,000 kg/ha of the biofertilizer could significantly reduce soil salt content, increase soil available nutrient contents, strengthen soil fertilizer-supplying capacity, improve crop stress resistance, and thus raise crop yield. The essence of biofertilizer is the synthetic microbial community that can rapidly activate indigenous beneficial microorganisms and raise the bacterial community diversity to develop as the dominant bacterial community in soil. This study further identified significant differential species through the comparison of the microbial communities between different treatments. Through RDA analysis, important soil nutrient factors significantly affecting the rice rhizosphere bacterial community structure and composition are discovered. It is indicated through PICRUSt function prediction that crop growth and crop yield have been promoted through increased metabolism of the bacteria community, membrane transport, and enzyme activity in soil by using the biofertilizer. In addition, these results provide a theoretical foundation for the improvement of microorganisms in the salinized field.

## Data availability statement

The datasets presented in this study can be found in online repositories. The names of the repository/repositories and accession number(s) can be found in the article/Supplementary material.

## Author contributions

DD and WC performed the study and wrote the original draft. DZ and GM designed the research and reviewed the manuscript. SS and ZW conducted data analysis and edited the manuscript. All authors contributed to the study and approved the submitted version.
