# Supplementary material for: Biofertilizer based on halotolerant microorganisms promotes the growth of rice plants and alleviates the effects of saline stress
Source: Front Microbiol. 2023 Jun 9;14:1165631. doi: 10.3389/fmicb.2023.1165631 (PMC10288287; doi:10.3389/fmicb.2023.1165631)
Supplement: Supplementary file 1 [file Table_1.docx]

**Supplementary TABLE 1** Information of the sequencing and field experimental samples

| **Original data number** | **Field experiment random number** |
| --- | --- |
| X_1 | CK1 |
| X_2 | CK3 |
| X_3 | GW1 |
| X_4 | GR1 |
| X_5 | CK2 |
| X_6 | GR3 |
| X_7 | GW2 |
| X_8 | GR2 |
| X_9 | GW3 |
